# NIR-triggerable self-assembly multifunctional nanocarriers to enhance the tumor penetration and photothermal therapy efficiency for castration-resistant prostate cancer

**DOI:** 10.1186/s11671-023-03802-y

**Published:** 2023-03-18

**Authors:** Shuqiang Li, Yan Ma, Chao Ma, Lei Shi, Fan Li, Liansheng Chang

**Affiliations:** 1 Department of Urology, The First Affiliated Hospital of Zhengzhou University, No. 1 Jianshe East Road, Zhengzhou, 450052 Henan China; 2 Department of Urology, Henan Cancer Hospital, No. 127 Dongming Road, Zhengzhou, 450003 Henan China

**Keywords:** Castration-resistant prostate cancer, Magnetic nanocarrier, Photothermal therapy

## Abstract

Great challenges still remain in the management of patients with castration-resistant prostate cancer (CRPC) based on traditional treatments, and the rapid development of nanotechnology may find a breakthrough. Herein, a novel type of multifunctional self-assembly magnetic nanocarriers (IR780-MNCs) containing iron oxide nanoparticles (Fe_3_O_4_ NPs) and IR780 iodide was synthesized by an optimized process. With a hydrodynamic diameter of 122 nm, a surface charge of –28.5 mV and the drug loading efficiency of 89.6%, IR780-MNCs have increased cellular uptake efficiency, long-term stability, ideal photothermal conversion ability and excellent superparamagnetic behavior. The in vitro study indicated that IR780-MNCs have excellent biocompatibility and could induce significant cell apoptosis under the 808 nm laser irradiation. The in vivo study showed that IR780-MNCs highly accumulated at the tumor area could reduce the tumor volume of tumor-bearing mice by 88.5% under the 808 nm laser irradiation, but minimal damage to surrounding normal tissues. Since IR780-MNCs encapsulated a large number of 10 nm homogeneous spherical Fe_3_O_4_ NPs, which can be used as *T*_2_ contrast agent, the best window for photothermal therapy can be determined through MRI. In conclusion, IR780-MNCs have initially showed excellent antitumor effect and biosafety in the treatment of CRPC. This work provides novel insights into the precise treatment of CRPC by using a safe nanoplatform based on the multifunctional nanocarriers.

## Introduction

Prostate cancer has become the most common malignant tumor in male genitourinary system with an increasing incidence globally. According to the report of global cancer statistics 2020, 1.4 million new cases of prostate cancer were estimated in the world, with an incidence (14.1%) only second to lung cancer (14.3%) in men [1]. Although great progress has been made in diagnostic technology [2], a considerable number of patients are still initially diagnosed as local advanced stage or metastasis due to its occult attack, leading to great challenges in the clinical diagnosis and treatment.

Meanwhile, in view of the high inter-cancer and intra-cancer heterogeneity, the prognosis of patients with either localized or metastatic prostate cancer varies widely among individuals [3, 4]. For the intermediate-risk and high-risk localized prostate cancer, patients still have a high risk of distant metastasis regardless of whether they have received local treatment before [5, 6]. Once metastasis occurs, androgen deprivation therapy (ADT) will become the standard systemic treatment to curb clinical progress [7]. Unfortunately, many patients will eventually progress to the lethal stage of androgen-independent prostate cancer, also called castration-resistant prostate cancer (CRPC), after a short period of good response [8].

Most notably, although the significant survival advantages of chemotherapy, novel AR-targeted agents, Poly ADP-ribose polymerase inhibitor and immune checkpoint inhibitors have been confirmed in CRPC [9–12], subsequent resistance occurs frequently in clinical treatment [13–15]. Furthermore, despite the effectiveness of combination therapy having been confirmed, the improvement of overall survival is still limited [16, 17]. In addition, the optimal therapeutic sequence remains unclear so far. To better predict the clinical efficacy and prognosis of patients with CRPC, genomic testing and nomograms have attracted more and more attentions [18–20]. However, it is still challenging to carry out widely in clinic. What’s more, the latest research has found that CRPC-like cells pre-existing in early primary prostate cancer can accelerate the acquisition of CRPC and do not depend on the implementation of ADT [21]. Therefore, exploring more ideal therapeutic measures to break the situation as soon as possible is an urgent problem to be solved.

Newer forms of cancer treatment, such as photothermal therapy (PTT) [22–24], photodynamic therapy [25], chemo-photothermal cancer therapy [26] and gene therapy [27], are explored to improve the therapeutic effect of drug-resistant tumors in recent decades [28]. Of which, PTT has the advantage of high efficiency, small trauma, and high selectivity, presenting high clinical significance. It has been primarily confirmed that nanoparticles contained iron oxide and IR780 could achieve good antitumor effect[29]. Furtherly, Rastinehad et al. have reported the initial results that laser-excited gold–silica nanoshells are feasible and safe in treating patients with low- or intermediate-risk localized prostate cancer [30]. As one kind of nanomaterials approved by the U.S. Food and Drug Administration, functionalized magnetic nanoparticles based on iron oxide have been widely used in tumor diagnosis and treatment due to their unique magnetic guidance characteristics [31], as well as their low-cost, good biocompatibility, excellent superparamagnetic behavior, high relaxation rate, and highly modifiable surface [32, 33]. IR780, with the improved ability at light penetration depth for deep tumor treatment, can convert light energy into a mass of heat under the excitation of 808 nm laser, heating the lesion up to the critical treatment temperature to kill the tumor cells, achieving the purpose of treating tumors [34–36]. Therefore, the synthesis of novel nanomaterials based on iron oxide NPs and IR780 is expected to bring new solutions for the treatment of CRPC.

**Scheme 1:**
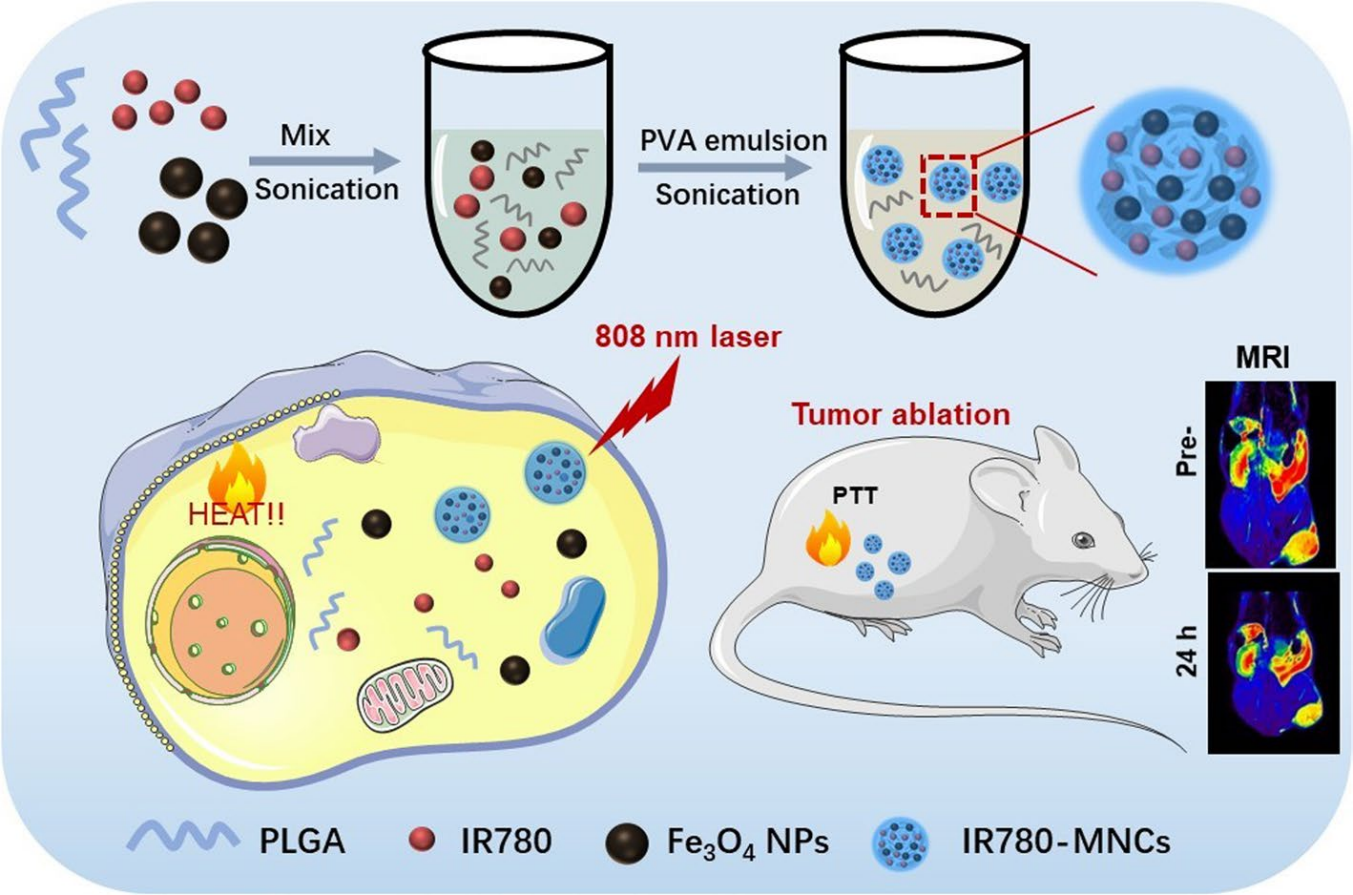
Schematic illustration of IR780-MNCs to enhance the tumor penetration and photothermal therapy efficiency for CRPC

Herein, we synthesized a kind of multifunctional IR780-triggerable self-assembly magnetic nanocarriers with ideal antitumor effect by fabricating with IR780 and Fe_3_O_4_ NPs through an optimized production process, named as IR780-MNCs, which can significantly extend the long-term stability during blood circulation and be enriched in prostate cancer cells via the enhanced permeability and retention effect (EPR). The biocompatibility and acid environment-controlled release property of IR780-MNCs endow their enormous clinical use in medical field of CRPC treatment. Particularly, the wrapped Fe_3_O_4_ NPs can be used as *T*_2_ contrast agents for magnetic resonance imaging (MRI) due to the long-term stability of IR780-MNCs in blood circulation, and the photosensitizer IR780 could generate continuous heat energy by converting from 808 nm laser irradiation, thereby inducing the cell apoptosis and tumor ablation. Our work found that the constructed IR780-MNCs can significantly extend the residence time in tumor region, reduce damage effects of drugs to the main organs, and finally achieve an ideal therapeutic effect in tumor-bearing mice (Scheme 1).

## Experimental section

### Materials and reagents

All analytically pure reagents were used as received without further purification. Iron (III) acetylacetonate, ethyl acetate, oleic acid, polyvinyl alcohol (PVA), chloroform, ethanol, oleylamine, benzyl ether, and dimethyl sulfoxide (DMSO) were ordered from Sinopharm Chemical Reagent Co., Ltd; PLGA, 1,2-hexadecanediol and IR780 were purchased from Sigma-Aldrich.

### Synthesis of Fe_3_O_4_ NPs

The monodisperse Fe_3_O_4_ NPs with a homogeneous size of 10 nm were obtained by the thermal decomposition method. 2 mmol of iron (III) acetylacetonate was mixed with 2 mmol of oleic acid, 2 mmol of oleylamine,10 mmol of 1,2-hexadecanediol and 10 mL of benzyl ether. Under magnetic stirring with the protection of N2 flow, the solution was firstly heated up to 200 °C for 2 h and further increased to reflux (~ 300 °C) for 1 h. Then, the obtained Fe_3_O_4_ NPs were precipitated and dispersed in CHCI3. The transmission electron microscopy (TEM, Thermo ScientificTM Talos F200X S/TEM) was used to observe their structures, and ImageJ was used for calculating their diameters. At last, the magnetic behavior of Fe_3_O_4_ NPs was identified by physical property measurement system (PPMS, DynaCool, USA), and the element composition was identified by the energy-dispersive X-ray spectroscopy (EDS) signal detection.

### Fabrication of IR780-MNCs

A mixture of IR780 (10 mg/mL in DMSO, 80 μg/mL), PLGA (2.5 mg/mL in ethyl acetate, 2.5 mL) and Fe_3_O_4_ NPs (20 mg/mL in chloroform, 100 μL) was sonicated for 20 min using a sonicator (180 W, Shanghai Juhong). Then, the mixed solution was dropwise added into 2 mL of PVA solution (2.5%, w/v) with 20 min sonication. Afterward, the scientz-IID ultrasonic cell disruptor (50 W, 10/10 s) was applied to carry out the fabrication of nanocarriers. During this procedure, both IR780 and Fe_3_O_4_ NPs were physically encapsulated into the polymer shell of PLGA molecules to obtain the homogeneous IR780-MNCs. At last, a rotary evaporator was used to evaporate the residual organic solvents and precipitate twice with centrifugation (6000 rcf, 10 min). The final IR780-MNCs were dispersed in Milli-Q water and stored at 4 °C. The structures and diameters of IR780-MNCs were analyzed by TEM and ImageJ.

### IR780 loading study

The absorption value of IR780 at 780 nm was acquired by UV–vis spectrophotometer (UV17 series, Shanghai Yoke Instrument Co., Ltd), and the obtained value is proportional to the concentration of added IR780. The IR780 loading efficiency (EE%) and IR780 loading content (%) were calculated with the following formula, separately:

(a) IR780 loading efficiency (%) = (Mass of loaded IR780)/(Total mass of IR780 added initially) × 100%;

(b) IR780 loading content (%) = (Mass of loaded IR780)/(Total mass of the final IR780-MNCs) × 100%.

The fluorescence spectra of IR780 and IR780-MNCs were recorded by fluorescence spectrophotometer (F320, Tianjin Gangdong SCI.&Tech. Co., Ltd., China) under the excitation wavelength at 700 nm. Besides, the hydrodynamic diameters and surface charges of IR780-MNCs were determined by Zetasizer Nano (Malvern Instruments Ltd., UK).

### Photothermal performance of IR780-MNCs

0.2 mL of IR780-MNCs with a series of concentrations (0.5, 1.0 and 1.5 mg/mL IR780) was added to the cell plate. The infrared thermal images with relative temperature were captured by an infrared thermal imaging camera (Testo, Germany) under 808 nm laser irradiation (1.0 W/cm^2^) for 5 min.

The IR780-MNCs were placed in the sodium citrate solution (50 mM, pH = 5.5) for 12 h, then half of them were irradiated with 808 nm laser (1.0 W/cm^2^, 5 min). Afterward, the sample was dropped in the copper grid for TEM measurement to investigate the morphological changes.

### Cellular uptake

To investigate the efficacy of IR780-MNCs in treating CRPC, DU145 cells were acquired from the Cell Bank of Cobioer (Nanjing, China). They were cultured in a high-glycemic DMEM medium containing 10% FBS, 100 U/mL penicillin and 0.1 mg/mL streptomycin under a humidified atmosphere of 37 °C and 5% CO_2_. DU145 cells (numbers: 4.0 × 10^4^) were cultured overnight in a 4-chamber glass-bottomed dish, IR780-MNCs (IR780 content: 5 µg/mL) were separately added and co-incubated for 2, 6, or 12 h. The cells were washed twice with PBS and further stained with DAPI for 10 min and fixed with 2.5% paraformaldehyde for 30 min. Afterward, the fluorescence signals were observed by a confocal laser scanning microscope (CLSM, TCS SP5II, Leica, Germany). Meanwhile, the fluorescence signals were also measured by flow cytometer (FCM, Attune NxT, Thermos Fisher, USA). Then, the scanning electron microscopy (SEM, GAIA3-TESCAN) and TEM (Tecnai G2, 120 kV) were used to capture the morphology of DU145 cells and the subcellular localization of IR780-MNCs.

### Cytotoxicity evaluation

Cell Counting Kit-8 (CCK-8) assay was performed to evaluate the dark toxicity (no laser treatment) and phototoxicity (808 nm laser treatment) of free IR780 and the IR780-MNCs on DU145 cell viability based on the changes of OD450 value recorded by Multiskan SkyHigh (Thermo Scientific, Germany). Cells incubated with PBS were used as control. 10^4^ cells/well of DU145 cells were cultured with serious concentrations of free IR780 and IR780-MNCs (5–25 μg/mL), respectively. After co-incubation for 24 h, the cells were washed twice by PBS solution and replaced with 100 μL of fresh cell medium. Afterward, the group for phototoxicity study was treated with an 808 nm laser for 5 min and cultured overnight. Then, 100 μL of CCK-8 solution (10%, v/v) in DMEM was added per well and incubated for another 4 h, and a standard CCK-8 assay was used to assess the cell viability by measuring the absorbance of each well at OD 450 nm using a Multiskan SkyHigh system. The calculation was following equation:

Cell viability (%) = (OD450 nm of experimental group)/(OD450 nm of control group) × 100%

Furthermore, FCM assays were used to investigate the cytotoxicity of PTT. After the cells were treated with different samples in the absence or presence of 808 nm laser irradiation (1.0 W/cm^2^, 5 min) and further incubated overnight, the cells were stained with Annexin V-FITC and PI to monitor the cell apoptosis rate by FCM. Besides, calcein AM/PI assay was used for the live/dead cell staining study of IR780-MNCs, after the cells incubated with different samples, a mixture solution of calcein AM (2.0 μM) and PI (1.5 μM) in PBS was added into each well, then the live cells stained by calcein AM as green fluorescence and dead cells stained by PI as red fluorescence were observed by CLSM.

### In vivo tumor-targeted MRI

Male BALB/c nude mice (ages: about 6 weeks; body weights: about 20 g) were used to construct tumor-bearing mouse models. The study was conducted according to the guidelines of the Declaration of Helsinki and approved by The Ethics Committee of Scientific Research and Clinical Trial of The First Affiliated Hospital of Zhengzhou University (Protocol code: 2022-KY-0197-001). In this work, all experimental mice were carefully raised in accordance with the guidelines provided by the Animal Protection and Use Committee. CRPC model was established by injecting 1 × 10^6^ DU145 cells into the right flank of mouse, when the average volume of tumor reached to about 100 mm^3^, those mice were used for the in vivo experiments.

Afterward, 3.0T nuclear magnetic resonance device (MAGNETOM skyra 3.0T, Siemens) was applied to evaluate the in vivo MRI of IR780-MNCs. IR780-MNCs (Fe content: 2.5 mg/kg) were intravenously injected into tumor-bearing mice, and in vivo *T*_*2*_-weighted MR images were captured over time (pre-injection, 0.5, 3, 9, 18, and 24 h). The relative pseudo-color images and MRI signal intensity were analyzed using ImageJ.

### In vivo antitumor efficacy of IR780-MNCs

During the in vivo experiment, tumor-bearing mice were randomized into six groups (n = 5), and the samples from different groups were intravenous injected for the PPT study: (1) PBS (as control), (2) free IR780 (content: 2.5 mg/kg), (3) IR780-MNCs (equivalent IR780: 2.5 mg/kg), (4) PBS + 808 nm laser (1.0 W/cm^2^, 5 min), (5) free IR780 + 808 nm laser (1.0 W/cm^2^, 5 min), (6) IR780-MNCs + 808 nm laser (1.0 W/cm^2^, 5 min). Among them, group (1–3) was considered as control, and group (4–6) was irradiated under 808 nm laser for 5 min at 24 and 48 h, respectively. Temperature changes were captured by an Infrared Thermal Imaging Camera, the body weight and tumor volume of mice were recorded per 3 days. Afterward, the mice were sacrificed on the 15th day post-injection, then their organ tissues and tumors were extracted carefully. Tumor volume was measured for antitumor efficacy analysis. TUNEL and hematoxylin–eosin (H&E) staining images were recorded to observe the morphological characteristics and pathological changes of the main organs and tumors.

### Statistical analyses

Prism software was used for statistical significance analysis. The signal indicated a significant difference (*: P < 0.01, **: P < 0.005, ***: P < 0.001).

## Results and discussion

### Synthesis of Fe_3_O_4_ NPs and IR780-MNCs

**Fig. 1:**
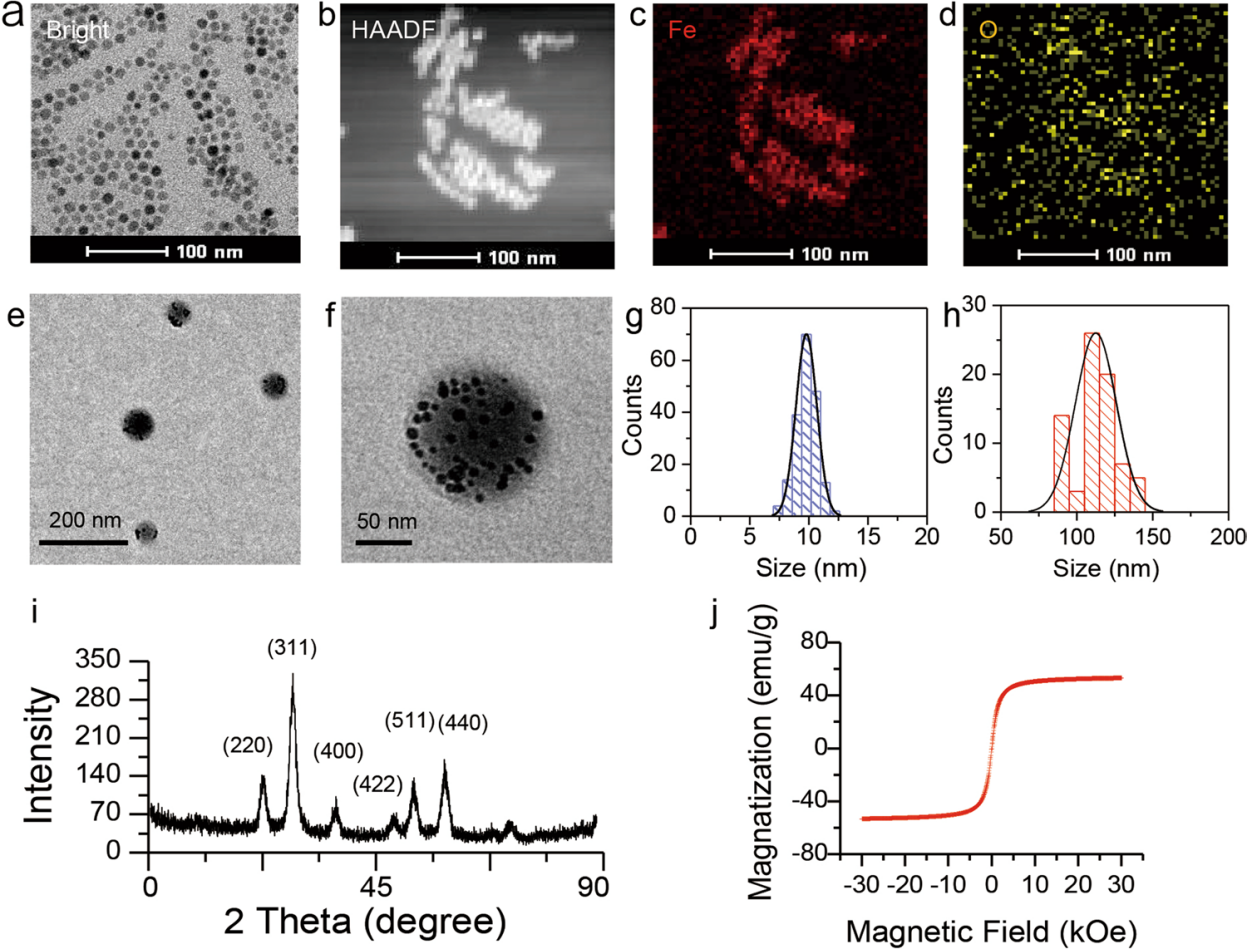
Synthesis and characterization of Fe_3_O_4_ NPs and IR780-MNCs. **a** Representative TEM photograph of homogeneous magnetic nanoparticles without any agglomerations, the sample dissolved in chloroform was dropped onto the ultra-thin copper film to visualize their morphology by transmission electron microcopy at 200 kV. **b–d** EDS element mapping photographs of magnetic nanoparticles (HAADF, elements of Fe and O, respectively). **e–f** Representative TEM photographs of self-assembly IR780-MNCs with uniform size, which was evaporated from water. **g–h** Size distribution histogram of Fe_3_O_4_ NPs and IR780-MNCs, the sizes collected by Image J were 10.3 ± 1.3 nm and 122.7 ± 8.1 nm, respectively. **(i)** X-ray diffraction pattern of Fe_3_O_4_ NPs, which was deposited on the glass substrates from chloroform. All the peaks are assigned at (220), (311), (440), (422), (511), and (440), which are consistent with the standard X-ray data. **(j)** Magnetic hysteresis loops (M-H) of Fe_3_O_4_ NPs measured at 300 K by PPMS

The monodisperse iron oxide magnetic nanoparticles (Fe_3_O_4_ NPs) were synthesized using a thermal decomposition strategy under a nitrogen flow atmosphere [37, 38]. As observed in Fig. 1a and g, Fe_3_O_4_ NPs displayed highly uniform spherical structure with an average diameter approximately 10 nm. Figure 1b–d shows the TEM photographs of high angle annular dark field (HAADF), indicating that Fe_3_O_4_ NPs contain Fe and O elements. Subsequently, IR780-MNCs were fabricated by Fe_3_O_4_ NPs and IR780, which exhibited homogeneous sizes with a large amount of Fe_3_O_4_ NPs inside (Fig. 1e–f). The relative percentage of Fe_3_O_4_ NPs loaded calculated by ICP-MS was 37.2%. The average diameter of IR780-MNC is approximately 122 nm (Fig. 1h). X-ray diffraction (XRD) in Fig. 1i further demonstrated that Fe_3_O_4_ NPs showed well-defined sharp diffraction peaks, particularly at 311, 220, and 440. This result indicated that our synthesized Fe_3_O_4_ NPs had good crystallinity. Besides, the magnetic behavior of Fe_3_O_4_ NPs was identified by physical property measurement system. Figure 1j shows that there is no hysteresis in the magnetic curve of synthesized Fe_3_O_4_ NPs measured at 300 K, and it can reach saturation magnetization immediately under a large external field, with the value of about 51.4 emu/g. This result demonstrated that our obtained Fe_3_O_4_ NPs have excellent superparamagnetic behavior.

### Physicochemical characterization

**Fig. 2:**
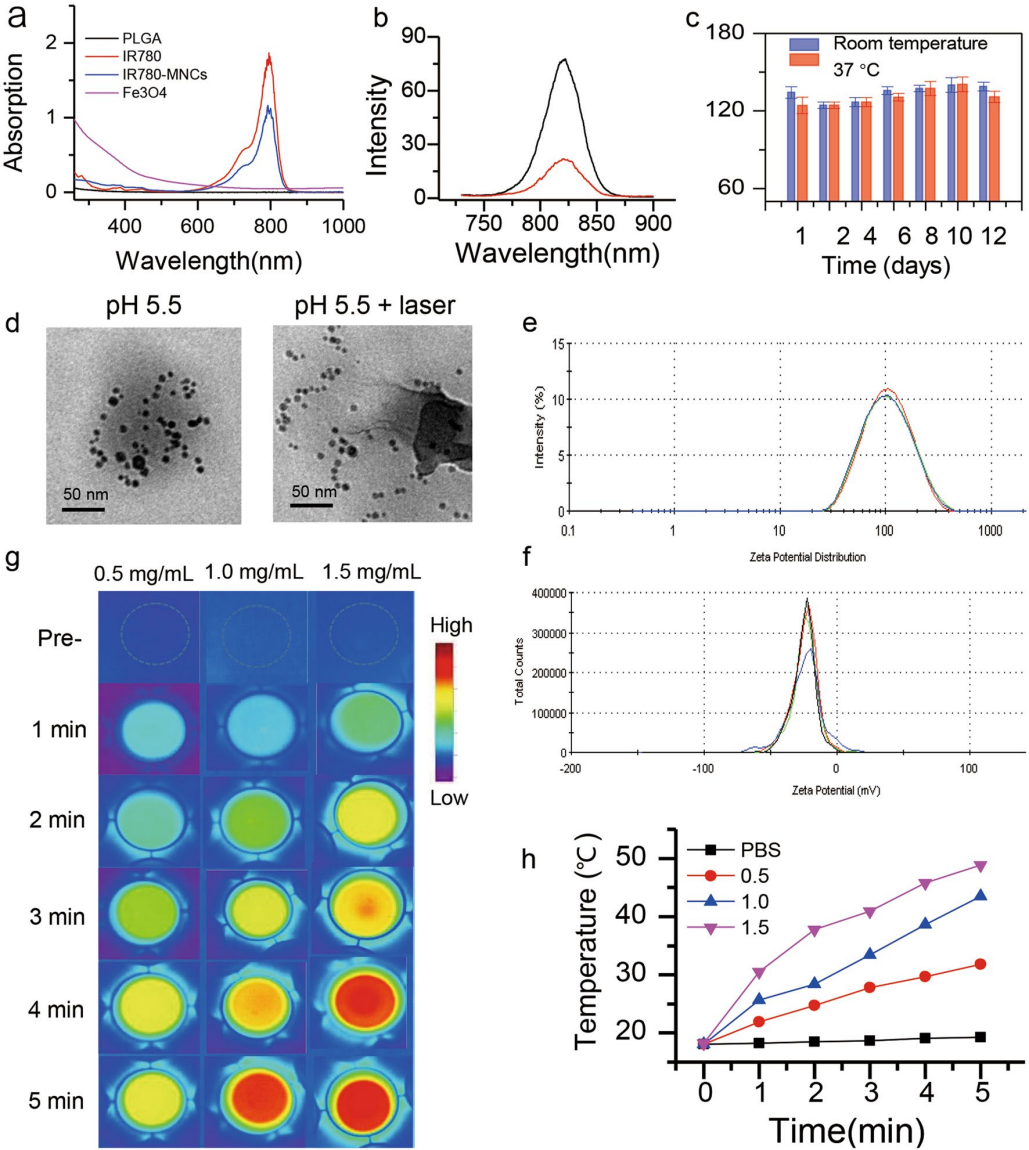
Fabrication and characterization of IR780-MNCs. **a** UV–Vis spectra of all the samples in the range of 230—1000 nm, such as Fe_3_O_4_ NPs in chloroform, PLGA in ethyl acetate, free IR780 in DMSO and IR780-MNCs in water. **b** The near-infrared (NIR) fluorescent spectra of free IR780 and IR780-MNCs in DMSO. The measured fluorescence peak was at 820 nm under the excitation wavelength at 700 nm. **c** The long-term stability evaluation of IR780-MNCs over a period of 12 days at 37 °C and room temperature, respectively. **d** Morphological changes of IR780-MNCs in acidic environment (pH 5.5) in the dark condition or treated with 808 nm laser irradiation. Scale bar: 50 nm. IR780-MNCs in neutral environment (PBS) was used as control group. **e** The hydrodynamic size distribution of IR780-MNCs dissolved in water (n = 3), with the analyzed hydrodynamic size was approximately 122 nm. **f** Zeta-potential value of IR780-MNCs dispersed in water (n = 3) with the corresponding value of approximately -28.5 mV. **g–h** Infrared thermal images and related temperature value of IR780-MNCs solution (0.5, 1.0 and 1.5 mg/mL) in the presence of 808 nm laser treatment (1.0 W/cm^2^, within 5 min)

To monitor the construction process of IR780-MNCs, UV–vis spectra of all the samples were recorded by a UV–vis spectrophotometer to analyze the absorption changes. As shown in Fig. 2a, Fe_3_O_4_ NPs dissolved in chloroform (rosy curve) have no evident absorption peak in the range of 230–1000 nm, and PLGA dispersed in ethyl acetate (black curve) shows almost a straight line without any absorption. In contrast, IR780 has a significant absorption peak at 798 nm in DMSO (red curve). After fabrication into IR780-MNCs, the typical absorption peak at 798 nm was clearly detected because of the strong absorption of IR780. Figure 2b shows the fluorescence spectra of IR780 and IR780-MNCs recorded by a fluorescence spectrophotometer, which presented a consistent emission peak at 820 nm under the excitation wavelength at 700 nm. Besides, the hydrodynamic diameters and surface charges of IR780-MNCs were determined by Zetasizer Nano, and the sizes of IR780-MNCs were approximately 122 nm described in Fig. 2e. The zeta-potential value was recorded as –28.5 mV (Fig. 2f), indicating that the fabricated IR780-MNCs are negatively charged nanosystem, which can prevent uptake from macrophages with longer lifetime of blood circulation than the positively charged ones [39]. Moreover, the long-term stability was investigated by monitoring the dynamic light scattering (DLS) sizes for 12 days. As shown in Fig. 2c, there is no significant DLS size changes of IR780-MNCs regardless of room temperature or 37 °C, demonstrating the highly stability in aqueous solution. However, the morphology of IR780-MNCs changes in acidic environment (pH 5.5) in the dark or under 808 nm laser irradiation, and IR780-MNCs placed in Milli-Q water was used as control group. As described in Fig. 2d, when the sample was dispersed in acidic solution (pH 5.5) for 12 h, irregular shape was recorded, indicating the acidic environment could affect the morphology of IR780-MNCs; when the sample in pH 5.5 solution was then irradiated with 808 nm laser (1.0 W/cm^2^) for 5 min, they showed apparently disintegrated, indicating that the 808 nm laser irradiation can accelerate the release of Fe_3_O_4_ NPs and IR780 in acidic environment.

Additionally, the optimal IR780 loading in the IR780-MNCs was evaluated, and the result showed that the drug loading efficiency is 89.6%, and drug loading content is 5.3%. Moreover, the infrared thermal images were used to evaluate the PTT conversion capability of IR780-MNCs under 808 nm laser irradiation. The photographs and relative temperature values were recorded by the Infrared Thermal Imaging Camera. As described in Fig. 2g-h, the initial temperature of the samples was 18.1 °C, in which the value was consistent with the room temperature. With the prolonging of 808 nm laser irradiation, the solution temperature increased rapidly, which is relying on the concentration of IR780. The temperature enhancement values (ΔT) of IR780-MNCs at 0.5, 1.0 and 1.5 mg/mL were 13.7, 25.4 and 30.7 °C, respectively. It has been proved that the photothermal conversion of nanomaterials can be used to treat a variety of tumors, including CRPC[40, 41]. Our result demonstrated that the novel synthesized IR780-MNCs maintain effective photothermal conversion ability, which could be used for PTT in treating CRPC.

### Cellular uptake assessment

**Fig. 3:**
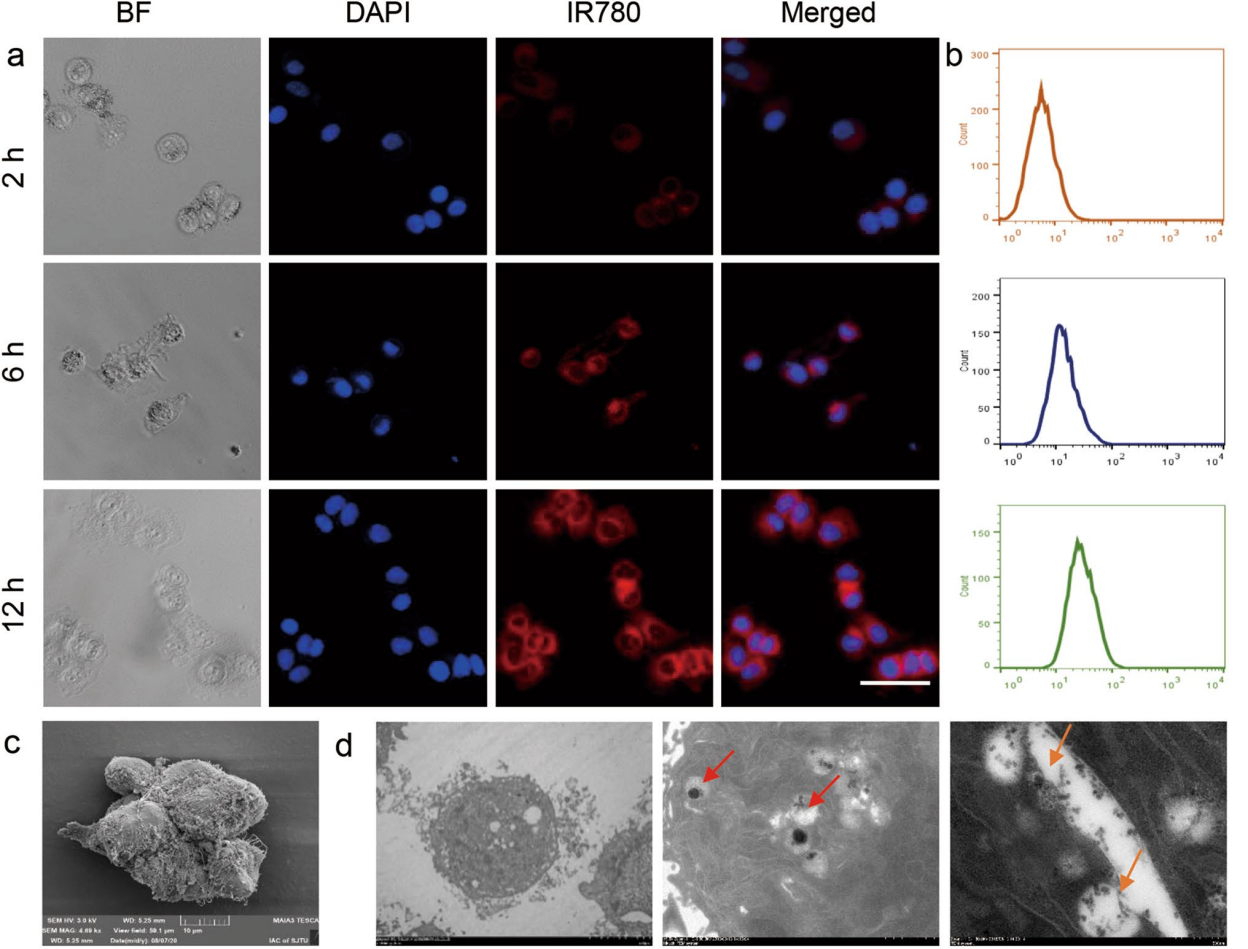
Cellular uptake analysis of IR780-MNCs. **a** Fluorescent signal images of prostate cancer cells captured by CLSM after incubated with IR780-MNCs for 2, 6 and 12 h, respectively. Cells without treatment were used as control group. IR780 concentration: 5 µg/mL. DAPI: blue channel (excitation wavelength: 405 nm, emission wavelength: 440–480 nm), and IR780: red channel (excitation wavelength: 633 nm, emission wavelength: 650–720 nm). Scale bar: 25 μm. **b** The relative cellular uptake intensity of IR780 collected by FCM. **c** SEM photograph of DU145 cells after incubation with IR780-MNCs, Scale bar: 10 μm. **d** Bio-TEM images of DU145 cells after incubation with IR780-MNCs, and different magnifications were recorded as below, in which the whole IR780-MNCs and free Fe_3_O_4_ NPs were clearly represented on the cell lysosome. Scale bar: 2, 5 μm and 500 nm

To evaluate the cellular uptake of IR780-MNCs in prostate cancer cells, a CLSM was used to record the fluorescence images of DU145 cells after cultured with IR780-MNCs, and a FCM was provided for collection of the relative cellular uptake intensity of IR780 molecules. Cells cultured with PBS were used as the control group. As shown in Fig. 3a–b, there was no obvious red fluorescence signal excited from IR780-MNCs cultured group in the first 2 h. When the incubation time was extended to 6 h, the red fluoresce signal was gradually enhanced and IR780-MNCs-treated cells presented higher fluorescence signal intensity than at 2 h. After 12 h of incubation, a stronger red fluorescent signal was detected, and the average signal of IR780-MNCs was about 1.5-folder higher compared with the one at 6 h, owing to the improvement of cellular uptake efficiency. Besides, the relevant Bio-SEM and TEM images of DU145 cells after cultured with IR780-MNCs were further captured using SEM and TEM, respectively. As shown in Fig. 3c, the surface morphology of prostate cancer cells was captured with a large number of tentacles observed on the surface of DU145 cells, which could help the cells ingest nanocarriers. In TEM images of cell cross section obtained in Fig. 3d, many IR780-MNCs with complete spherical structures were mainly agglomerated in cell lysosomes (red arrow), and meanwhile, a large amount of Fe_3_O_4_ NPs sustainably released from the ruptured IR780-MNCs were also clearly represented (yellow arrow). This result demonstrated that IR780-MNCs could be efficiently ingested and delivered into the lysosomes of DU145 cells and gradually degraded in the acidic environment in the cancer cells.

### In vitro antitumor effect

**Fig. 4:**
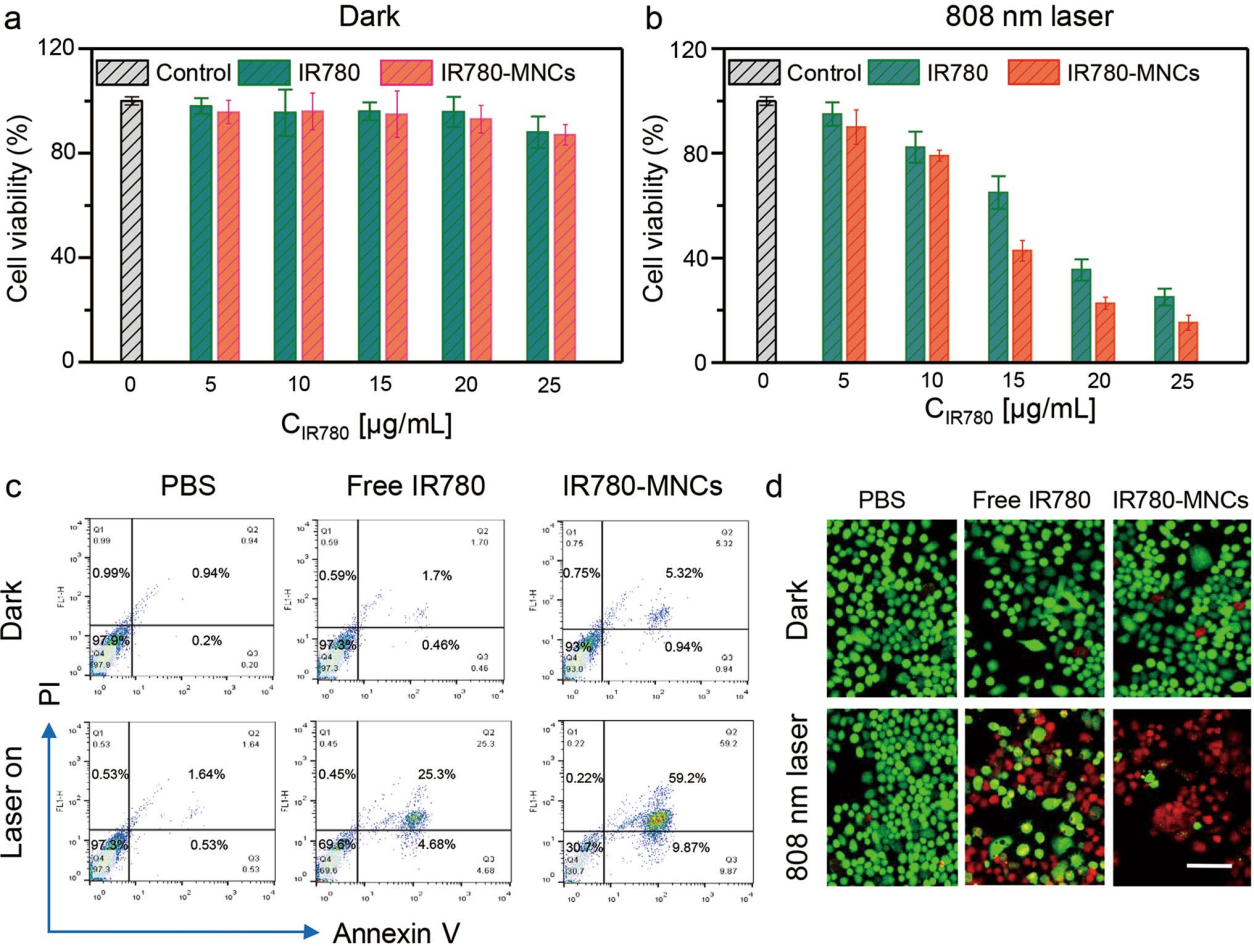
In vitro antitumor evaluation of IR780-MNCs. **a, b** Cellular viability assessment of DU145 cells co-exposed with different concentrations of free IR780 and IR780-MNCs (IR780 content: 0–25 µg/mL). The assessment was obtained by CCK-8 assay with or without 808 nm laser irradiation (1.0 W/cm^2^, 5 min). **c** The relative cell apoptotic rates of different samples treated with or without 808 nm laser treatment, respectively. **d** The calcein AM/PI staining assessment of prostate cancer cells after treated with PBS, free IR780 and IR780-MNCs, respectively. Scale bar: 50 μm

The cytotoxicity effect was evaluated by CCK-8 assay in DU145 cells through recording the absorbance at OD 450 nm using Multiskan SkyHigh, and the cells treated with PBS were set as the control group. As shown in Fig. 4a, due to the excellent biocompatibility of IR780 molecules, there was no obvious cell death observed after incubated with free IR780 and IR780-MNCs at the series of concentrations (IR780 content: 0–25 μg/mL) in the dark treatment. They remained a high cell viability about 86.5% even rich to the content of 25 μg/mL, indicating the good biocompatibility of free IR780 and IR780-MNCs, whereas after the treatment with 808 nm laser (1.0 W/cm^2^) for 5 min, the obvious cell death was observed after co-exposure with free IR780 or IR780-MNCs, and this appearance is relying on the increase in IR780 concentration. As shown in Fig. 4b, the cell viability values were decreased from about 90% to 20% when the IR780 concentration increased from 5 to 25 µg/mL. This result is mainly because the IR780 taken up by DU145 cells could transform into enough heat with the treatment of 808 nm laser, inducing the cell apoptosis and accelerating their death. Additionally, the cells were labeled with PI/Annexin V-FITC to investigate the cell cytotoxicity of IR780-MNCs using FCM. As shown in Fig. 4c, no laser treatment will not induce distinct cell death, but the 808 nm laser irradiation could significantly induce cell cytotoxicity incubated with IR780 or IR780-MNCs, respectively. Furthermore, the calcein AM/PI staining was used to study the photodamage effect of IR780-MNCs after different treatments (Fig. 4d), all the samples showed almost all green spots with dark conditions, demonstrating that all the cells are alive; in comparison, red spots as dead cells were observed in the group of the IR780 or IR780-MNCs in the presence of 808 nm laser. This result is consistent with the study mentioned above. Therefore, IR780-MNCs have effective photothermal effects, further confirming their promising potentials for CRPC treatment.

### MRI-guided PTT treatment of IR780-MNCs

**Fig. 5:**
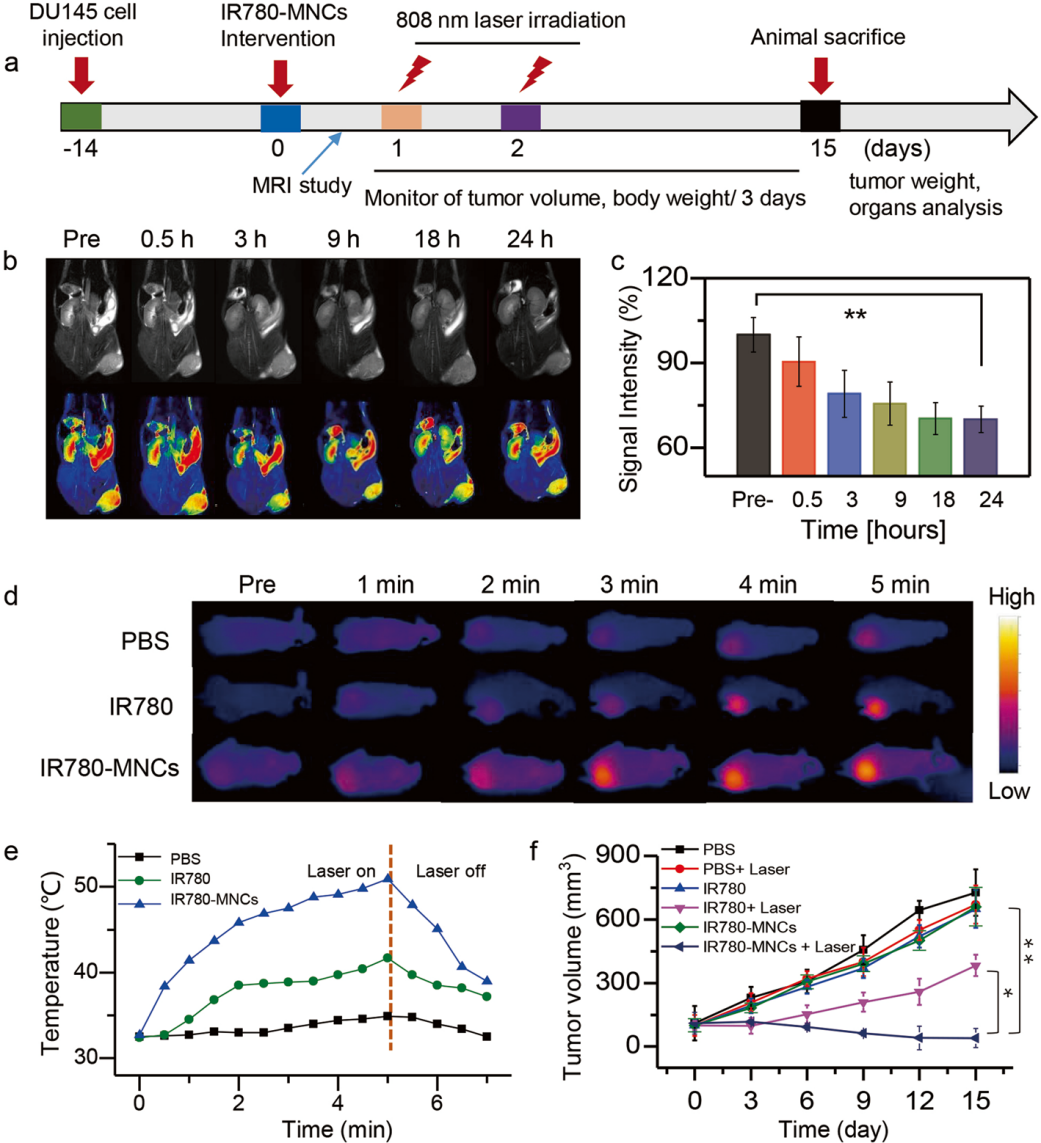
MRI-guided PTT treatment of IR780-MNCs. **a** The schematic diagram of in vivo experiment. **b** *T*_*2*_-weighted MRI images with the relative pseudo-color images of prostate-tumor-bearing mice captured using a 3.0 T nuclear magnetic resonance device. The pictures were recorded before injection (Pre-) or after intravenous injection of IR780-MNCs (0.5, 3, 9, 18 and 24 h). **c** The corresponding MRI signal values at the tumor region were analyzed using software Image J (n = 3). **d–e** Infrared thermal images of prostate-tumor-bearing mice and the representative temperature–time changes at the tumor site were recorded by infrared thermal device. The free IR780 and IR780-MNCs with IR780 content of 2.5 mg/kg, as well as PBS, were intravenous injected into the prostate-tumor-bearing mice in different groups, and the temperature changes were monitored after 808 nm laser on (1.0 W/cm^2^, 5 min) or laser off. **(f)** The variation in tumor volume curves of DU145 tumor-bearing mice from different groups 15 days after intravenous injection

MRI-guided PTT treatment of IR780-MNCs was then evaluated in the prostate-tumor-bearing mice. The schematic diagram of in vivo experiment is shown in Fig. 5a, the MR imaging in vivo was used to study the tumor enrichment ability of IR780-MNCs and window of the optimal PTT treatment. After intravenous injection of IR780-MNCs in tumor-bearing mice, Fe_3_O_4_ NPs wrapped in IR780-MNCs were used as *T*_*2*_ contrast agent, and relative MRI photographs and pseudo-color images were recorded over time (pre-injection, 0.5, 3, 9, 18, and 24 h) using a 3.0 T nuclear magnetic resonance device. After collecting MRI signals through the tumor areas, their relative values were then analyzed using Image J to evaluate the enrichment efficiency of IR780-MNCs. The mice before injection were used as the control group. As represented in Fig. 5b–c, the contrast at tumor region gradually turns dark, and the relative MRI intensity value at 24 h is about 55% lower than that of pre-injection. This demonstrated that the IR780-MNCs gradually enrich to the tumor area with the systemic blood circulation and lead to a decrease in MRI value and obvious darkness of the tumor part at 24 h post-injection.

To further investigate the near-infrared (NIR)-induced high-efficiency thermal damage and tumor accumulation of IR780-MNCs, infrared thermal images of prostate-tumor-bearing mice were recorded in Fig. 5d. 808 nm laser (1.0 W/cm^2^) was irradiated at the tumor parts for 5 min at 24 and 48 h, respectively. The mice without any treatment were used as the control group. Figure 5e shows that the initial temperature at tumor area was approximately 32.5 °C before any laser treatments. When an 808 nm laser exposed to the tumor site for 5 min, the PBS-treated mice have no obvious temperature rise at tumor region, the temperature difference (ΔT) is about 3 °C, indicating that the safe power of 808 nm laser without any tissue damages. However, the mice treated with free IR780 or IR780-MNCs had varying degrees of temperature rise, with the temperature at tumor site reached to maximum of 41.6 and 50.9 °C, but minimal damage to surrounding normal tissues. Thus, IR780-MNCs-treated mice had efficient PTT effect because of the high accumulation of IR780 at the tumor area. Therefore, our fabricated IR780-MNCs have effective PTT ability to induce cell necrosis.

Furtherly, the relative tumor volumes of prostate-tumor-bearing mice were monitored to assess the PTT therapeutic effect after treatment with PBS, free IR780 or IR780-MNCs, respectively. Figure 5f shows that very little antitumor effect was observed in all the control groups without 808 nm laser irradiation, and the maximum tumor volume was about 690 mm^3^. In comparison, the groups treated with free IR780 + 808 nm laser had significant inhibition of tumor growth, and the tumor volume was reduced to 50%, showing a moderate anticancer outcome. The best antitumor effect was observed in the IR780-MNCs + 808 nm laser group, in which 88.5% tumor reduction was achieved compared with the control group. This result demonstrated that the synthesized IR780-MNCs in our study have excellent PTT therapeutic effect with 808 nm exposure.

### In vivo biocompatibility assessment of IR780-MNCs

**Fig. 6:**
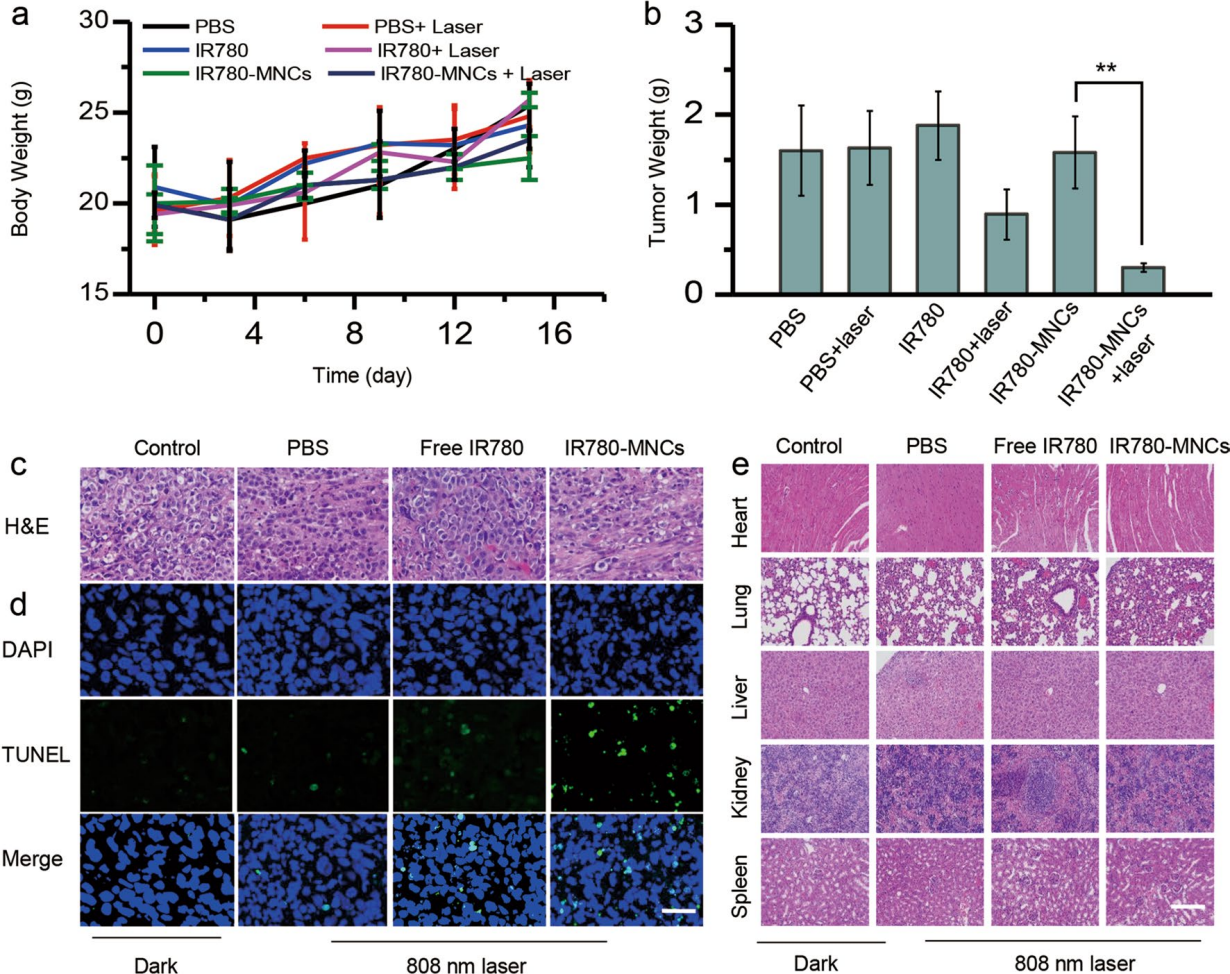
The analysis of ex vivo organs and tumors. **a** The body weight growth curves of DU145 tumor-bearing mice from different groups of 15 days. **b** Tumor weights from different groups after various treatment at day 15 (n = 5). **c, d** H&E staining and TUNEL images of tumor tissue from different groups, including PBS, free IR780 and IR780-MNCs-treated mice after 808 nm laser treatment for 15 days. **e** H&E staining images of heart, lung, liver, kidney and spleen after intravenous injections under dark condition or 808 nm laser irradiation after 15 days. Scale bar: 100 μm

The body weight growth curves of tumor-bearing mice from different groups of 15 days showed that there was no evident weight loss during the PTT (Fig. 6a). After 15 days of observation, the mice were killed, and the main organs and tumors were harvested for further analysis. As shown in Fig. 6b, both IR780 and IR780-MNCs-treated groups had significant lower tumor weight, and the weight vales from IR780-MNCs-treated mice decreased 80% compared with the control group. They were further analyzed by H&E staining and TUNEL displayed in Fig. 6c–d, evident green fluorescence had detected from IR780-MNCs-treated mice under 808 nm laser irradiation, indicating more tumor cell apoptosis. This is because IR780-MNCs have enhanced tumor permeability and long-term retention, thus showing excellent treatment efficiency. Additionally, the biocompatibility of IR780-MNCs was carefully evaluated in the prostate-tumor-bearing mice. Then, various organs (including heart, lung, liver, kidney, and spleen) from different groups were dissociated and H&E staining was further performed to evaluate the potential toxicity of IR780-MNCs to tumor-bearing mice. As displayed in Fig. 6e, under the dark conditions or NIR laser irradiation, H&E staining of these main organs’ sections confirmed that there was no obvious damage or abnormality during 15-day treatment. Therefore, all the results indicated that our designed IR780-MNCs have minimal short-term toxicity that can be used as biosafe therapeutics for further clinical applications.

## Conclusions

In summary, the novel multifunctional IR780-MNCs based on PLGA molecules containing magnetic Fe_3_O_4_ NPs and IR780 were fabricated via microemulsion strategy to improve the delivery efficiency of prostate tumor during photothermal treatment. The biocompatibility, long-term stability and acid environment-controlled release performances of IR780-MNCs endow their enormous clinical use in medical study for the prostate cancer. These nanosystems could significantly prolong the blood circulation period and efficiently accumulate in the tumor site by EPR effect in the DU145 tumor-bearing mice. Moreover, the wrapped Fe_3_O_4_ NPs and IR780 were gradually released from IR780-MNCs in an acidic environment due to the effect of cell lysosome and 808 nm laser irradiation and further penetrated into the deep area because of the small size for enhanced anticancer performance in prostate-tumor-bearing mice. Fe_3_O_4_ NPs as *T*_2_ contrast agent could achieve the real-time MR imaging over time and further confirm the accumulation time at tumor area for precise cancer treatment. Furthermore, under the 808 nm laser irradiation, the released IR780 could generate a large amount of heat for photothermal therapy. In vitro and in vivo antitumor studies confirmed that IR780-MNCs had excellent photothermal therapeutic efficacy and antitumor effects on prostate cancer when compared with the control groups. Therefore, this study provides a novel strategy to overcome the clinical treatment dilemma of drug-resistant prostate cancer, which is expected to be used in a wide range of clinical applications for CRPC in the near future.

## Data Availability

The datasets used and/or analyzed during the current study are available from the corresponding author on reasonable request.

## Abbreviations

CRPC: Castration-resistant prostate cancer
Fe_3_O_4_ NPs: Iron oxide nanoparticles
PLGA: Poly lactic-co-glycolic acid
IR780-MNCs: IR780-triggerable self-assembly magnetic nanocarriers
EPR: Enhanced permeability and retention effect
MRI: Magnetic resonance imaging
DMSO: Dimethyl sulfoxide
TEM: Transmission electron microcopy
CLSM: Confocal laser scanning microscope
FCM: Flow cytometer
SEM: Scanning electron microscopy
CCK-8: Cell counting kit-8
PTT: Photothermal therapy
H&E: Hematoxylin–eosin
